# Evaluating microbial load on dental curing lights and the impact of protective barriers on resin composites

**DOI:** 10.1590/1807-3107bor-2025.vol39.085

**Published:** 2025-09-08

**Authors:** Maria Eugênia ALVAREZ-LEITE, Flávia Araújo ALVES, Adrielly Ferreira CARDOZO, Pedro Aleixo Garcia Paim RIBEIRO, Victor de Morais GOMES, Cristina Dutra VIEIRA, Márcia Almeida LANA, Alberto Nogueira da Gama ANTUNES

**Affiliations:** (a) Pontifícia Universidade Católica de Minas Gerais – PUC-Minas, Institute of Biological and Health Sciences, Dentistry Department, Belo Horizonte, MG, Brasil.; (b) Newton Paiva Ferreira Cultural Institute, School of Dentistry, Department of Restorative Dentistry, Belo Horizonte, MG, Brazil.; (c) Universidade Federal de Minas Gerais – UFMG, Institute of Biological Sciences, Department of Microbiology, Belo Horizonte, MG, Brasil.

**Keywords:** Disinfection, Curing Lights, Dental, Dentistry, Decontamination, Infection Control, Dental

## Abstract

The contamination of dental curing light tips was evaluated before and after treatment and after their use and disinfection. The influence of a plastic protective barrier over the flexural strength and the modulus of elasticity of resin composites were also analyzed. Microbiological sampling was conducted at initial contamination (T0), in Log 10 CFU/4 mL; after dental treatment (T1); and after disinfection with 70% ethanol (v/v) (T2). The results were analyzed by descriptive statistics and analysis of variance. The flexural strength and the modulus of elasticity analyses were performed using bar-shaped test specimens of three commercial resin composites with and without barriers, and the tests were subjected to a statistical normality test. Turbidity was observed in the media in 60.7% of the tubes at T0, 100.0% at T1, and 57.1% at T2. The microbial contamination was similar at T0 and T2, but a significant increase was observed at T1. The recovered microbial load differed significantly between T1 and T2 (p < 0.05). The results of the flexural strength and modulus of elasticity analyses showed no significant differences with or without a barrier for any of the different colors of resin, polymerization time, or the three resins. Under the present experimental conditions, 70% ethanol significantly reduced the levels of microbial contamination, but it did not guarantee the inactivation of all microbial cells. The use of plastic protective barriers did not alter the flexural strength or the modulus of elasticity of any of the tested resin composites, indicating that they are a safe and viable measure to prevent cross-contamination when using a dental curing light.

## Introduction

Aerosols produced during dental care by slow- and high-speed handpieces, three-way syringes, and scalers contaminate the clinical environment through airborne microorganisms.^1,2^ These microorganisms can be inhaled by both the patient and clinical staff, increasing the risk of spreading respiratory diseases such as COVID-19.^3^ In dental healthcare services, blood and saliva may be a source of contamination on environmental surfaces, affecting patients, dental professionals, and any device that may be in contact with those surfaces.^4^ For this reason, equipment and surfaces (especially those that are difficult to clean) should be protected with some form of barrier in order to prevent contamination.^5^ Following each patient’s appointment or after an item has been used, these barriers (plastic wraps or other materials impervious to moisture) have to be removed and discarded. If the surfaces cannot be covered and become contaminated, they have to be cleaned and subsequently disinfected with intermediate- or high-level chemical solutions.^1^

Dental curing lights are used in resin composite restorations,^6^ and hardening these composites is a crucial process that occurs through the conversion of monomers into polymers.^7^ Resin composite is routinely used in dentistry,^8^ and so are dental curing lights.^9^ As curing lights are constantly being used and shared and are impossible to sterilize, they are a potential source of cross-contamination. Despite the biological risk, dental students and professionals often neglect proper processing of dental curing lights to prevent contamination. Although rarely discussed, this lack of compliance with established guidelines is frequent and the most common reason seems to be the fear of interfering with the process of resin composite polymerization. Furthermore, as dental restorations are not an invasive procedure, it is believed that the risk of cross-contamination is lower.

Disinfection should guarantee a surface free of elements that may increase the risk of infection^5^ and safeguard the efficacy of restorative treatment. The use of protective barriers may be considered a practical method for preventing contamination.^1^ However, it is not known precisely to what extent a plastic barrier might interfere with the polymerization process, especially when some distance already exists between the curing light tip and the resin composite. Resin composite fillings have the goal of restoring the aesthetics and function of a tooth. Their longevity and clinical success rely directly on proper polymerization, given that curing mechanisms act upon the physical and mechanical properties of the resin.^10^ It is crucial to maintain the efficacy of dental curing lights and to minimize the risk of cross-contamination.^11^ Bearing this in mind, the goal of this study was to quantitatively and qualitatively evaluate the microbial load of dental curing light tips before and after dental procedures and after the final disinfection. Additionally, the influence of protective barriers on the flexural strength and modulus of elasticity of commercial brands of resin composite were evaluated. Finally, the present study has two null hypotheses (H_0_) to be confirmed: disinfecting dental curing light tips with 70% ethanol (v/v) for one minute may not be able to effectively eliminate the entire microbial load; and using a protective plastic barrier would not interfere with the flexural strength and the modulus of elasticity of resin composites.

## Methods

### Analysis of microbial contamination of dental curing lights

Microbiological evaluation was carried out at the Laboratory of Microbiology of the Life Sciences and Health Institute at PUC Minas (ICBS – PUC Minas), Belo Horizonte, Minas Gerais, Brazil. Twenty-nine dental curing light tips were analyzed, all from the clinics at the Department of Dentistry of PUC Minas, at three different time points: T0: before use; T1: after the clinical procedure; and T2: after chemical disinfection. Three trained dental technicians who provided assistance to all dental clinics performed the disinfection procedure. The disinfection technique followed the university’s protocol. The photopolymerization unit was wiped with 70% alcohol (v/v) for one minute after use and before storage. After the clinical procedure and before disinfection, the equipment was wrapped in disposable plastic bags and stored until the next use. The sampling area was defined as extending 1 cm from the active tip of the device. The active tip touches the tooth surface, oral fluids, and mucosa during resin composite restoration procedures and polymerization. Sample collection took place at T0, T1, and T2 through sterile swab friction against the tip area under aseptic conditions. The swab was moistened with sterile saline solution (0.85%) and slowly and firmly rubbed for one minute.

### Processing the clinical specimens: quantitative and qualitative microbiological evaluation

The specimens were transported and processed in sterile and hermetically sealed stainless-steel packaging. The first sample collection (T0) measured the initial level of contamination. Subsequently, sampling was repeated after the dental procedure and before the disinfection process in order to evaluate the microbial load (T1). Finally, the last sample collection occurred after tip disinfection (T2). The chemical solution used for disinfection consisted of 70% ethanol (v/v), which was rubbed on the selected area for one minute. For the qualitative evaluation, the swabs were immersed in 4 mL of brain heart infusion broth (BHI) (Difco Laboratories, Detroit, USA) and then vortexed for 60 seconds.^8,12^ The tubes were incubated at 37 ºC for 48 hours under aerobic conditions. The final results were noted as the number of colony-forming units (CFUs), indicating the microbial load in 4 mL (CFU/4 mL). The qualitative evaluation was based on the presence or absence and the level of turbidity. When positive, the level of turbidity was visually recorded and ranged from + (scarcely grown) to +++ (heavily grown). After measuring’ microbial turbidity, a 0.1 mL aliquot was inoculated in BHI agar in duplicate and incubated at 37ºC for 48 hours under aerobic conditions. The cultures were then counted, and their morphotypes were recorded and analyzed.

### Evaluating the influence of a protective barrier on the flexural strength and modulus of elasticity of resin composites

#### Production of test specimens of resin composites

**Figure 1:**
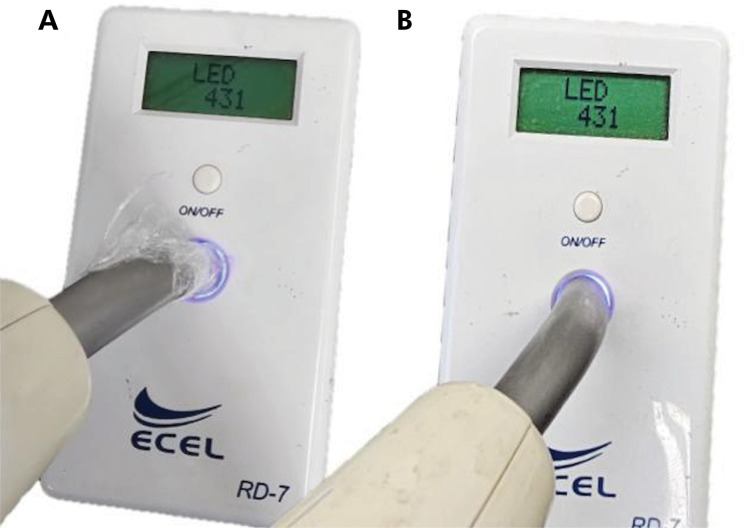
RD7 radiometer measurement of the intensity of light curing with and without the use of a protective barrier.

Flexural strength and modulus of elasticity tests were carried out at the Laboratory of Engineering of PUC Minas, Belo Horizonte, Minas Gerais, Brazil. For the two experimental conditions (with and without use of protective barriers), 10 bar-shaped test specimens measuring 12 mm x 2 mm x 2 mm molded in a medium-rigidity rubber container (ODEME-Dental Research, Luzerna, Brazil) were made. The size of the bar-shaped test was smaller than that recommended by the International Organization for Standardization in an attempt to simulate clinical procedures in dental practice.^13,14^ The protective barriers consisted of a 9 µm-thick translucent polyvinyl chlorine (PVC) film, designed for easy removal after application (Banfilme Bandeirante Indústria e Comércio de Plásticos Ltda, São Paulo, Brazil). The central region of each test specimen was polymerized according to Table 1. The exposure time was selected considering whether the barrier could interfere with a low polymerization power. The curing times of 20 and 40 seconds were used to evaluate if they could improve the mechanical properties of the resin composites. The values of light intensity also simulated a lower exposure time to the curing light to detect a potentially negative effect of the protective barrier. Polymerization was performed using an ECEL device, model SPACELED (RD 7, ECEL Indústria e Comércio Ltda, Ribeirão Preto, Brazil), available at the university’s dental clinic. The irradiance of the light emitting diode (LED) was 431 mW/cm^2^, as specified by the manufacturer. The values were measured with the RD 7 radiometer (RD 7, ECEL Indústria e Comércio Ltda, Ribeirão Preto, Brazil) (Figure 1).

**Table 1:** Details about the tested resin composites, considering light-curing exposure time, shade number, and manufacturer.

Resin composite	Shade number	Light-curing exposure (in seconds)	Manufacturer
Forma	2A2 enamel	20	ULTRADENT
Forma	2A2 dentin	20	ULTRADENT
Z100 A2	2A2	40	3M ESPE, USA
Tetric N Ceram	2A2 enamel	40	IVOCLAR VIVADENT AG - LIECHTENSTEIN
Tetric N Ceram	EA2 dentin	20	IVOCLAR VIVADENT AG - LIECHTENSTEIN

**Figure 2:**
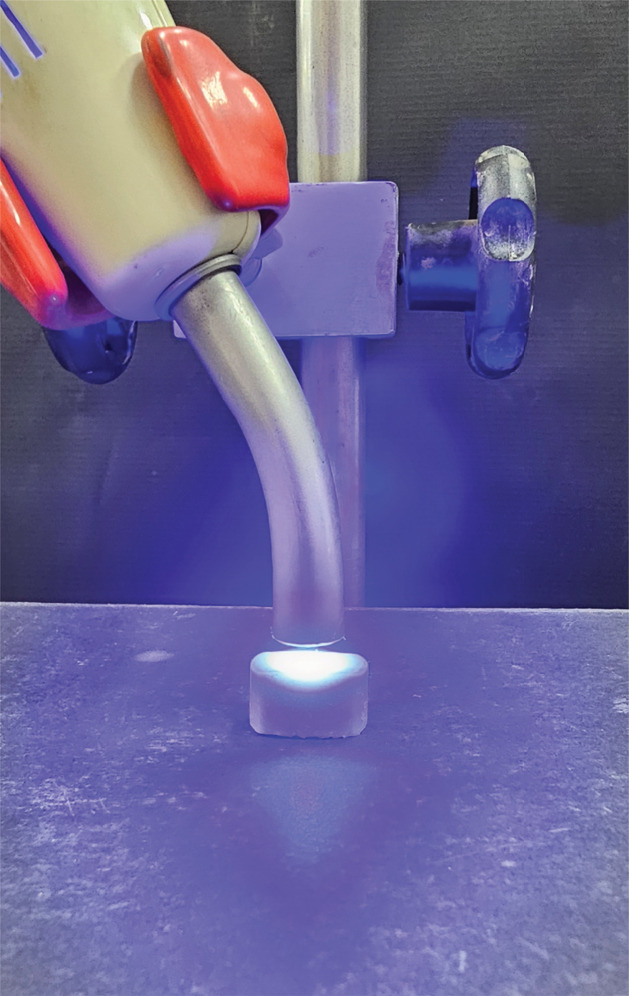
Curing light tip mounted on a metallic support during the experiments.

During light curing, the curing light tip (11 mm) was mounted on a metallic support (Figure 2) at a distance of 5 mm from the outermost surface of the resin composite, which had been inserted in the rubber mold in one portion. The distance between the supports was 10 mm. The selected distance was an attempt to simulate dental clinical procedures in which the tip of the LED device does not directly touch the surface of the resin composites, such as the gingival wall of class II cavities. Ten specimens were prepared for each testing condition in Table 1 using a protective barrier wrapped around the curing light tip and 10 specimens without the barrier, totaling 100 resin composite bars.

## Measuring the flexural strength and the modulus of elasticity of resin composites

The bar-shaped test specimens (n = 10) of resin composite were subjected to a three-point flexural test on a universal testing machine (EMIC 23-5D, Paraná, Brazil) at a crosshead speed of 0.5 mm/minute and cell load of 500N.^15,16^ Each test specimen was placed at the center of the test fixture on the machine, with the specimen surface that was closest to the light source facing up. The values for flexural strength and elastic modulus were calculated according to the formulas:

a.Flexural strength (calculated in megapascals (MPa):


$$\sigma_{f}=3FL/2wd^{2}$$

[F = fracture load in newtons (N); L = span length (mm); w = sample width (mm); d = sample thickness (mm)].

b.Modulus of elasticity (calculated in GPa):


$$E_{f}=FL^{3}/4wd^{3}D$$

[F = fracture load in newtons (N); L = span length (mm); w = sample width (mm); d = sample thickness (mm); D = sample deflection (mm)].

After fracture, the values for the bar-shaped test specimens were recorded in MPa and GPa. The flexural strength and the modulus of elasticity conditions used herein were similar to those in other studies that used mini-flexural testing considered slightly similar to a clinical scenario.^13, 14^

## Statistical analysis

The microbial load recovered from dental curing lights was described by descriptive statistics and analysis of variance (two-way ANOVA; GraphPad Prism 7 Software, USA). The results of flexural strength and modulus of elasticity tests were subjected to a normality test using the same program (GraphPad Prism 7 Software, USA).

## Results

### Analysis of the microbial load of dental curing lights

**Figure 3:**
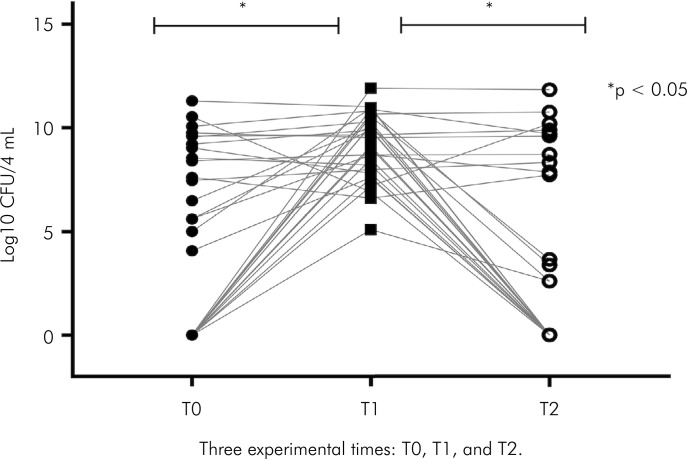
Quantitative evaluation of microbial load (Log10 CFU/4 mL) of dental curing lights at three different time points: before (T0) and after (T1) dental procedures, and after dental treatment and surface disinfection (T2).

The results for the quantitative microbial tests are shown in Table 2 and Figure 3.

**Table 2:** Quantitative evaluation of microbial load (in Log 10 CFU/4 mL) of dental curing lights before (T0) and after (T1) dental procedures, and after dental treatment and surface disinfection (T2).

Curing light test number	Microbial load (Log 10 CFU/4 mL)		
	T0	T1	T2
1	9.6	9.7	9.9
2	6.5	10.0	2.6
3	10.0	10.8	3.4
4	0	9.1	0
5	8.5	8.2	0
6	0	10.5	3.6
7	0	11.9	11.8
9	0	5.0	2.6
10	0	10.9	9.7
11	5.6	9.9	0
12	4.0	7.6	0
13	0	10.1	0
14	0	10.7	10.8
15	5	10.9	0
16	9.5	10.3	0
17	7.5	7.9	8.3
18	0	7.6	0
19	9.2	9.9	0
20	0	9.0	0
21	0	8.5	0
22	8.4	8.7	7.9
23	0	7.1	10.2
24	10.5	6.9	0
26	11.3	11.0	0
28	5.6	8.7	8.7
30	9.8	9.5	9.6
31	7.6	6.6	7.7
34	0	10.1	0
Mean	4.8	9.1	3.7
Standard deviation	4.3	10.8	4.5

*p<0.05. Each dot, circle, or square represents a single curing light contamination at a given test time (Log 10 CFU/4 mL). The lines connecting the dots, circles, and squares show the recorded values for each tested device throughout the experiment.

The average contamination level at T0 was 4.8±4.4 (Log10 CFU/4 mL). After use in a procedure (T1), the average level of contamination of dental curing lights was 9.2±10.8 (Log10 CFU/4 mL). At T2, the average level of contamination was 3.7±4.5 (Log10 CFU/4 mL). The level of microbial contamination was similar before the procedure (T0) and after disinfection (T2). On the other hand, we found a significant increase in the number of CFUs on dental curing lights immediately after the dental procedures. Interestingly, a statistically significant (p < 0.05) reduction in the number of bacterial colonies was observed when comparing T1 (after the dental procedure) and T2 (after the device had been disinfected) (Figure 1).

In five tests (17.4%), post-procedure disinfection (T2) could not reduce the levels of microbial load (Log10 CFU/4 mL) compared to T1. The average reduction in contamination after disinfection was 55% (in Log 10 CFU/4 mL, SD ± 43%).

The qualitative evaluation based on the absence or presence of turbidity in the growth media (Table 3) showed contamination in 60.7% of the tubes at T0, while at T1 there was growth in all broth media. After disinfection of the tips (T2) with 70% ethanol (v/v), we observed microbial growth in over half of the tubes (57.1%).

**Table 3:** Qualitative evaluation of microbial load (level of turbidity) on dental curing lights before (T0) and after dental procedures (T1), and after dental treatment and surface disinfection (T2).

Curing light	Visual qualitative evaluation		
	T0	T1	T2
1	+++	+++	+++
2	+	+++	+++
3	+++	+++	+
4	-	+++	-
5	++	+++	-
6	-	++	+
7	-	+++	+++
9	-	+	+
10	-	+++	+++
11	+	+++	+
12	-	+	-
13	-	++	-
14		++	++
15	+	++	-
16	+	++	-
17	+	+	+
18	-	+	-
19	+	+	-
20	+	+	-
22	+	++	-
23	-	++	++
24	++	++	+
26	++	++	-
28	+	+	+
30	+	+	+
31	+	+	+
34	-	+	++
36	+	+	-
Presence of turbidity n (%)	17/28 (60.7%)	28/28 (100%)	16/28 (57.1%)

(-) absence of turbidity; = absence of microbial growth; (+) presence of turbidity = presence of microbial growth; +: scarcely grown; ++: moderately growth; +++: heavily grown.

### Evaluating the influence of a protective barrier on the flexural strength and elastic modulus of resin composites.

Table 4 shows the values of flexural strength, in MPa, for the resin composite test bar from the brand Forma, both with and without a protective barrier. It also shows the modulus of elasticity in GPa of the same commercial brand. We found no significant differences between the values of flexural strength (p = 0.4) and modulus of elasticity (p = 0.5) when using or not using a plastic protective barrier under the test conditions, regardless of resin composite color or curing time. The exposure curing time of 10 seconds was tested during the experiments and resulted in insufficient polymerization of the bar-shaped test specimens.

**Table 4:** Mean and standard deviation of flexural strength (in MPa) and of modulus of elasticity (in GPa) for test bar specimens produced with Forma resin composite without and with a plastic protective barrier.

Resin composite / curing time	Flexural strength (MPa)^*^		Modulus of elasticity (GPa)^**^	
	Protective barrier			
	Without	With	Without	With
Forma enamel/ 20 seconds	178.1 (25.7)^a^	175.6 (14.5) ^b^	2.2 (0.2)^e^	2.6 (0.5)^f^
Forma enamel/ 40 seconds	173.1 (7.2) ^a^	182.9 (26.1) ^b^	2.5 (0.4)^e^	2.8 (0.5) ^f^
Forma dentin/ 20 seconds	176.9 (30.9)^c^	164.2 (21.7)^d^	2.38 (0.3)^g^	2.2 (0.5)^h^
Forma dentin/ 40 seconds	167.4 (19.5) ^c^	187.8 (11.3) ^d^	2.3 (0.5)^g^	2.9 (0.3)^h^

*Two-way ANOVA with significance level of 95%. p-value = 0.4; **Two-way ANOVA with significance level of 95%. p-value = 0.5.

^a^p-value obtained during flexural strength analysis (MPa) on Forma enamel without a barrier showed no statistical significance; ^b^p-value obtained during flexural strength analysis (MPa) on Forma enamel with a barrier showed no statistical significance; ^c^p-value obtained during flexural strength analysis (MPa) on Forma dentin without a barrier showed no statistical significance; ^d^p-value obtained during flexural strength analysis (MPa) on Forma dentin with a barrier showed no statistical significance; ^e^p-value obtained during modulus of elasticity analysis (GPa) on Forma enamel without a barrier showed no statistical significance; ^f^p-value obtained during modulus of elasticity analysis (GPa) on Forma enamel with a barrier showed no statistical significance; ^g^p-value obtained during modulus of elasticity analysis (GPa) on Forma dentin without a barrier showed no statistical significance; ^h^p-value obtained during modulus of elasticity analysis (GPa) on Forma dentin with a barrier showed no statistical significance.

The Tetric N-Ceram resin composite was subjected to the same tests, and the results are shown in Table 5. We also found no significant differences between the flexural strength (p = 0.4) and modulus of elasticity (p = 0.6) when using or not using a plastic protective barrier under the tested conditions, regardless of resin composite color and curing time.

**Table 5:** Mean and standard deviation of flexural strength (in MPa) and of modulus of elasticity (in GPa) for test bar specimens produced with Tetric Ceram resin composites, without and with a plastic protective barrier.

Resin composite / curing time (in seconds)	Flexural strength (MPa)*		Modulus of elasticity (GPa)^***^	
	Protective barrier			
	Without	With	Without	With
Tetric enamel/ 20 s	155.1 (16.1)^a^	168.5 (8.8)^b^	2.2 (0.2)^e^	2.2 (0.3)^f^
Tetric enamel/ 40 s	176.9 (24.2) ^a^	166.4 (13.9)^b^	2.1 (0.3)^e^	2.3 (0.3)^f^
Tetric dentin/ 20 s	152.7 (14.2) ^c^	164.9 (12.1)^d^	1.8 (0.6)^g^	2.2 (0.2)^h^
Tetric dentin/ 40 s	158.0 (16.1) ^c^	153.2 (14.9)^d^	2.0 (0.4)^g^	2.1 (0.2)^h^

*Two-way ANOVA with significance level of 95%. p-value = 0.4; **^2^Two-way ANOVA with significance level of 95%. p-value = 0.6. ^a^p-values obtained during flexural strength analysis (MPa) on Tetric enamel without a barrier showed no statistical significance. ^b^p-values obtained during flexural strength analysis (MPa) on Tetric enamel with a barrier showed no statistical significance; ^c^p-value obtained during flexural strength analysis (MPa) on Tetric dentin without a barrier showed no statistical significance; ^d^p-value obtained during flexural strength analysis (MPa) on Tetric dentin with a barrier showed no statistical significance; ^e^p-values obtained during modulus of elasticity analysis (GPa) on Tetric enamel without a barrier showed no statistical significance; ^f^p-value obtained during modulus of elasticity analysis (GPa) on Tetric enamel with a barrier showed no statistical significance; ^g^p-value obtained during modulus of elasticity analysis (GPa) on Tetric dentin without a barrier showed no statistical significance; ^h^p-value obtained during modulus of elasticity analysis (GPa) on Tetric dentin with a barrier showed no statistical significance.

Table 6 shows the data for the test bar specimens made with the Z100 resin composite. Similarly to the other two resin composites, there were no significant differences in the flexural strength (p = 0.2) or the modulus of elasticity (p = 0.3). It is important to highlight that this resin composite did not have different colors for the different structures of the teeth (enamel and dentin).

**Table 6:** Mean and standard deviation of flexural strength (in MPa) and of modulus of elasticity (in GPa) for test bar specimens produced with Z100 resin composite, without and with a plastic protective barrier.

Resin Composite/ curing time (in seconds)	Flexural strength (MPa)*		Modulus of elasticity (GPa)^**^	
	Protective barrier			
	Without	With	Without	With
Z100/ 20 s	182.4 (22.3)^a^	177.0 (21.9)^b^	2.9 (0.3)^c^	2.8 (0.4)^d^
Z100/ 40 s	192.6 (21.0)^a^	183.4 (28.1)^b^	3.1 (0.7)^c^	2.8 (0.8)^d^

*Two-way ANOVA with significance level of 95%. p-value = 0.2. **Two-way ANOVA with significance level of 95%. p-value = 0.3. ^a^p-values obtained during flexural strength analysis (MPa) on Z100 without a barrier showed no statistical significance; ^b^p-value obtained during flexural strength analysis (MPa) on Z100 with a barrier showed no statistical significance; ^c^p-values obtained during modulus of elasticity analysis (GPa) on Z100 without a barrier showed no statistical significance; ^d^p-value obtained during modulus of elasticity analysis (GPa) on Z100 with a barrier showed no statistical significance.

## Discussion

Dental curing lights are routinely used in most clinical dental procedures.^9^ Most of the time, these devices come into contact with oral fluids. In some clinical situations, curing lights are occasionally in direct and also indirect contact with blood through hands and gloves. Therefore, they are classified as semicritical instruments.^5^ Semicritical items are those that come in contact with mucous membranes or non-intact skin.^5,17^ These devices should be free from any microorganisms, although small numbers of bacterial spores may be present.^15^ Considering that these instruments are semicritical and have the potential to create cross-contamination, the current recommendation is to disinfect them using, preferably, high-level chemical agents.^3,4^ According to the literature,^18^ disinfection is an essential measure that halts the dissemination of all infectious agents by inactivating them and preventing their transmission. In this study, 70% ethanol (v/v), a chemical solution classified as intermediate-level, was used. According to the Centers for Diseases Control,^5^ intermediate-level disinfectants might be biocidal for mycobacteria, vegetative bacteria, most viruses, and most fungi, but they do not necessarily kill bacterial spores. Despite that, the recommendation of the 70% ethanol (v/v) use in clinical protocols and its use in dental college departments^5,19,20^was taken into account.

This study evaluated the effect of disinfecting dental curing lights. The overwhelming majority of items tested showed a reduction in the microbial load following disinfection (Tables 2 and 3; Figure 3). It was also observed that contamination remained detectable in over half of the curing lights. The qualitative evaluation (Table 3) was more sensitive than the quantitative one (Table 2), but did not provide a precise numerical evaluation of the level of decontamination. According to the CDC, the evaluation of microbial contamination from devices is essential to observe the effects of an infection-control practice. The obtained results should be carefully analyzed and could be used for future comparisons.^21^ The results of the quantitative study showed a statistically significant reduction in microbial load after clinical use and disinfection (Figure 3). Nonetheless, there was no difference between the levels of contamination before the clinical procedure and after disinfection with 70% ethanol (v/v). It is important to highlight that over half of the tested items still had the potential risk for microbial contamination after disinfection. They still presented a potential microbial load sufficient to cause contamination, although this was reduced in comparison to the levels detected immediately after the procedure (Figure 3). Accordingly, the first hypothesis of the present study was confirmed (H_0_) because disinfection procedures did not guarantee the destruction of all microbial cells. While internationally recommended,^21^ disinfection with 70% ethanol requires the absence of organic matter and a rigorous friction technique to be effective.^22^ These criteria could not be routinely followed owing to characteristics of clinical dental practice in different institutions. The literature specifically discussing the contamination and disinfection of dental curing lights is scarce, but the obtained results corroborate the findings from similar scientific studies that used the same disinfection agent and other similar items in dentistry.^23^

The efficacy of disinfection with 70% ethanol (v/v) varies according to the microorganism tested, the conditions of the disinfected item (presence or absence of visible dirtiness), and the technique used.^24^ Variation may also occur depending on the care taken while cleaning the items. A study measured the efficacy of four chemical disinfectants and found that S. mutans cells were viable for up to 60 minutes of immersion in 70% ethanol (v/v). The authors also found bacterial granules (formed by agglutination of microbial cells), and these structures potentially increased the viability of the bacteria even after 60 minutes of immersion in an alcohol solution.^25^

Single-use protective barriers that cover potentially contaminable surfaces and are discarded after a clinical procedure are a powerful precaution to prevent cross-contamination among the dental staff and patients.^5,26^ However, the possibility of protective barriers interfering with the efficacy of resin composite polymerization is considered an obstacle to their use,^11^ especially when there is a considerable distance between the curing light tip and the resin itself. The results on the interference of protective barriers during the use of dental curing lights on three different composite resins (Forma, Tetric Ceram, and Z100) did not show any significant differences in the flexural strength or modulus of elasticity (Tables 4 through 6) when using or not using protective barriers. The finding confirmed the second hypothesis of the present study (H_0_), as no changes were detected in the tested resin composites. The study by Soares et al.^11^ analyzed the effect of using barriers correctly or incorrectly on different parameters of light curing units. The authors demonstrated that different plastic barriers could reduce from 5% to 26% of the light output (mW), especially when incorrectly used. Despite the differences in methodology, they found that the PVC protective barrier presented the lowest value of interference with photopolymerization devices,^11^ the same barrier tested in the present study. Soares et al.^11^ also emphasized that curing light barriers are an important cross-infection routine to be adopted between clinical procedures. No significant differences were observed in this study regarding the translucency of materials. This property is defined by Salgado et al.^27^ as one of the most important optical aspects to consider when evaluating aesthetics. Translucency results from the relationship between the refractive indices of the filler particles and the resin matrix.^27^ Notwithstanding the different translucency values, enamel- and dentin-colored resins yielded the same results (Table 6). These findings suggest that the use of a physical protective barrier is a practical and simple measure to prevent and control cross-contamination. Because it is an external method of protection, any microbial contamination that might arise from the use of the curing light active tip would remain on top of the barrier itself, and should be removed at the end of the treatment and appropriately discarded after the procedure. Therefore, these findings indicate that the physical barrier is an internationally recommended^21^ option for protecting hard-to-clean equipment surfaces.^19^ No significant reduction was observed in microbial load between T0 and T2, suggesting that protective barriers could be more effective than disinfection with 70% ethanol (v/v), considering cross-infection control protocols. Using a protective barrier in dental curing lights is a simple and effective procedure^21^ and should be included in institutional protocols.

The present study has some limitations. Firstly, the storage conditions for the disinfectant solution were not recorded. The environmental temperature and the performance of the disinfection process itself, among other variables, were also not evaluated. All these aspects could reduce the efficacy of the chemical solution. Such aspects also support the hypothesis that the variability observed in microbial growth after disinfection may be even greater in routine clinical settings. Secondly, this study only evaluated the occurrence of bacteria under aerobic conditions. If anaerobic incubation had been possible, we believe that the results would have been even more interesting. Despite causing a significant reduction in microbial load after dental procedures, disinfection with ethanol may not guarantee the absence of viable microbial cells, thus not preventing cross-contamination altogether. Thirdly, the experimental conditions did not include cleaning prior to 70% ethanol disinfection.

## Conclusion

Under the tested conditions, disinfecting dental curing light tips using 70% ethanol (v/v) significantly reduced the levels of contamination after their use in clinical procedures. These findings confirm the first hypothesis of this study, as disinfection procedures did not guarantee the destruction of all microbial cells. Covering dental curing lights with protective barriers such as plastic films did not significantly interfere with the flexural strength or the modulus of elasticity of the tested resin composites. These findings also confirmed the second hypothesis, given that no alterations were detected in the tested resin composites. Therefore, this measure stands out as a viable and safer alternative for the prevention of cross-contamination from dental curing lights.

## Data Availability

The authors declare that all data generated or analyzed during this study are included in this published article.
